# High-throughput sequencing enhanced phage display enables the identification of patient-specific epitope motifs in serum

**DOI:** 10.1038/srep12913

**Published:** 2015-08-06

**Authors:** Anders Christiansen, Jens V. Kringelum, Christian S. Hansen, Katrine L. Bøgh, Eric Sullivan, Jigar Patel, Neil M. Rigby, Thomas Eiwegger, Zsolt Szépfalusi, Federico de Masi, Morten Nielsen, Ole Lund, Martin Dufva

**Affiliations:** 1 Department of Micro- and Nanotechnology, Technical University of Denmark, Kgs. Lyngby, Denmark; 2 Center for Biological Sequence Analysis, Technical University of Denmark, Kgs. Lyngby, Denmark; 3 National Food Institute, Technical University of Denmark, Søborg, Denmark; 4 Roche NimbleGen, Madison, Wisconsin, the United States of America; 5 Institute of Food Research, Norwich, United Kingdom; 6 Department of Paediatrics, Medical University of Vienna, Vienna, Austria; 7 Instituto de Investigaciones Biotecnológicas, Universidad Nacional de San Martín, Buenos Aires, Argentina

## Abstract

Phage display is a prominent screening technique with a multitude of applications including therapeutic antibody development and mapping of antigen epitopes. In this study, phages were selected based on their interaction with patient serum and exhaustively characterised by high-throughput sequencing. A bioinformatics approach was developed in order to identify peptide motifs of interest based on clustering and contrasting to control samples. Comparison of patient and control samples confirmed a major issue in phage display, namely the selection of unspecific peptides. The potential of the bioinformatic approach was demonstrated by identifying epitopes of a prominent peanut allergen, Ara h 1, in sera from patients with severe peanut allergy. The identified epitopes were confirmed by high-density peptide micro-arrays. The present study demonstrates that high-throughput sequencing can empower phage display by (i) enabling the analysis of complex biological samples, (ii) circumventing the traditional laborious picking and functional testing of individual phage clones and (iii) reducing the number of selection rounds.

The concept of phage display was originally introduced in 1985^1^ and is still one of the predominant techniques for the screening of protein-protein interactions. Phage display is based on libraries of phage particles expressing a great variety of exogenous peptides fused to phage surface proteins. The highly diverse library is reduced to a few leads by performing several rounds of selection, also known as biopanning^2^,^3^,^4^,^5^. Phage display has been widely applied in antibody discovery and has given rise to several therapeutic antibodies^6^,^7^,^8^. A range of different types of phage libraries have been constructed, with display of short random peptides or antibody domains as prominent examples^2^.

Phage display has been extensively used for the identification of antibody binding sites on antigens, known as epitope mapping^2^,^9^. Identification of epitopes is an important step in the development of diagnostic tools, in rational vaccine design and in identification of therapeutic targets^10^,^11^,^12^,^13^. Epitopes are generally classified as being either linear (continuous) or conformational (discontinuous). Whereas linear epitopes comprise short stretches of consecutive amino acid residues of the primary antigen sequence, conformational epitopes consist of residues that are distant in the primary sequence, but are brought close by the conformational folding of the native protein^9^.

A prominent issue in phage display is the unintended selection of peptides that do not bind the target of interest, collectively known as “target unrelated peptides” (TUPs)^14^,^15^. Generally, TUPs either bind a constant part of the screening platform or provide the phage with a proliferation advantage. Constant parts of the screening platform include the solid phase (such as plastic or beads) as well as constant parts of antibodies or other capturing molecules^14^,^15^. A routine step in phage display is the amplification in bacteria. During this process, a pool of phages propagates in parallel and even minor proliferation advantages can have a profound impact on the output after this step. As a consequence, library diversity is shaped by selection as well as amplification, as thoroughly discussed by Derda *et al.*^16^. One way to deal with the TUP issue is to compare phage clones isolated in an experiment with databases encompassing previously isolated peptides and known TUP motifs^17^,^18^.

Today, the emergence of high-throughput sequencing techniques^19^ is transforming multiple areas (e.g. immune repertoire characterization^20^) and is well suited for analysis of the inherent diversity of phage libraries. The data output from deep sequencing should enable the analysis of highly complex samples by phage display and holds the potential to circumvent the traditional laborious picking and functional testing of individual phage clones. Accordingly, in recent years there have been considerable efforts in coupling high-throughput sequencing with phage display. Initially, the potential of this approach was demonstrated by studying protein interactions in a high-throughput manner^21^,^22^,^23^ followed by studies of antibody display libraries^24^,^25^,^26^,^27^,^28^. Recently, a gene-specific library was used to identify immunodominant regions of a vaccine antigen^29^. With regard to random peptide display libraries, an initial study that examined the overlap in peptides obtained by conventional versus high-throughput sequencing coupled phage display^30^ has been followed by a few others. One study used deep sequencing to assist the identification of TUP candidates with a propagation advantage^31^. Two other studies employed high-throughput sequencing to identify peptides that bound to cell surfaces^32^,^33^. However, even though a major application of phage display is in epitope mapping, until now the potential of using high-throughput sequencing enhanced phage display for epitope mapping has only been described to a limited degree. Ryvkin *et al.* demonstrated that a fraction of the peptides obtained after binding to IgG from a pool of HIV positive sera could be aligned to an HIV protein thus indicating some HIV specificity^34^. Liu *et al.* took a broader approach and searched for putative IgG binding targets using BLASTP to determine the similarity between isolated phage peptides and all known proteins^35^. They established the approach on immunised mice as well as a sample from a human melanoma patient. These studies have been very limited with regards to the amount of human patient material and suggested epitopes have not been validated. Nonetheless, these studies encourage further investigations of the potential of high-throughput sequencing-assisted epitope mapping directly on serum.

In this study, we exploit the enormous data output of high-throughput sequencing to investigate samples of great complexity, specifically serum samples from patients with severe peanut allergy. Peanut allergy is regarded as one of the most serious forms of food allergy, in terms of prevalence, persistency and severity^36^,^37^ and Ara h 1, which is the focus of this study, is one of the major peanut allergens^36^,^37^,^38^. We present a general approach to address the issues of TUPs, identify patient-specific epitope motifs directly from serum, and validate identified epitopes using high-density peptide micro-arrays.

## Results

### Deep sequencing of phage libraries selected against patient serum

**Figure 1:**
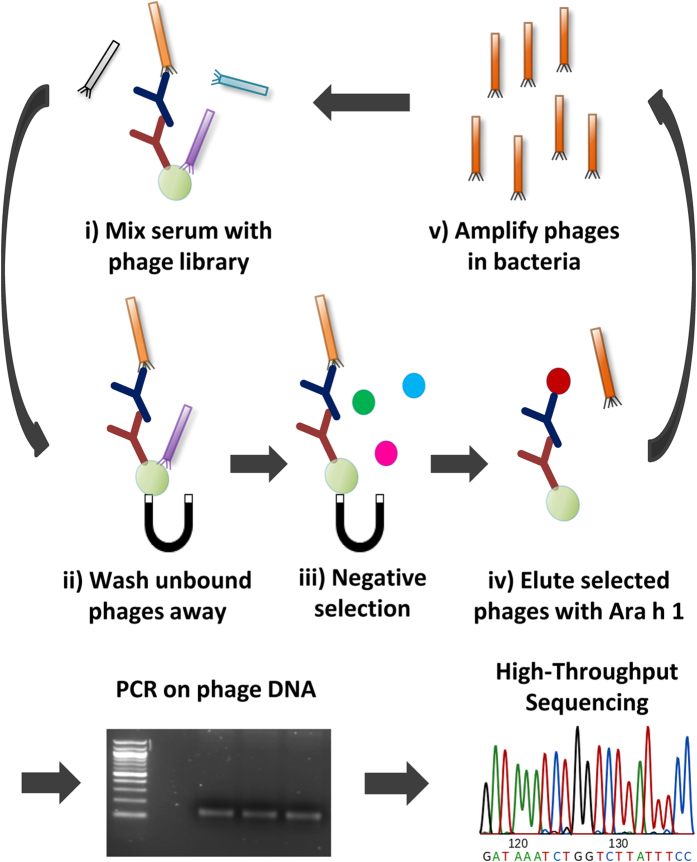
An overview of the phage selection process. (**i**) The IgE from serum is captured by beads coated with anti-IgE. The phage library is added and (**ii**) unbound phages are washed away. (**iii**) Negative selection, specifically incubation with SMP, is carried out to minimise unspecific binding. (**iv**) Phages expressing epitope mimicking peptides are eluted by competitive addition of Ara h 1. (**v**) The eluted phages are amplified in bacteria and either used for another selection round (starting at (**i**) again) or subjected to PCR and high-throughput sequencing.

The phage selection process (see Fig. 1) was based on the Ph.D.7^TM^ phage library, which displays 7-mer peptides. This library has a reported diversity of 10^9^, approaching the theoretical diversity (1.28 × 10^9^), thus covering the majority of possible amino acid combinations. The phage particles were selected over 3 rounds of biopanning against IgE from 12 subject samples, comprising both patients with severe peanut allergy as well as control subjects with no reactivity towards peanut allergens. Specifically, the samples were sera from 4 patients (P1-4) and 4 controls (C1-4) as well as plasma from 2 of these patients (P1 and P4) and 2 of the controls (C2p and C3p). In order to investigate whether there were changes in the epitope patterns over time, the plasma samples from the 2 patients (P1_12 and P4_12) were obtained about 9 years after the serum samples (P1 and P4). Epitope mimicking peptides were selected by competitive addition of Ara h 1. Following PCR amplification the phage DNA was sequenced by high-throughput sequencing. For every one of the 12 samples, DNA was obtained for all 3 selection rounds and subjected to deep sequencing.

For each of the 36 sampled conditions an average of 308,235 (standard deviation (sd) = 102,719) DNA sequences were obtained. Of these, an average of 231,642 (sd = 104,649) could be converted to peptide sequences. Identified peptides were further processed by removing peptides originating from DNA sequences with low quality and not having the NNK codon structure encoded in the phage library. Due to the high prevalence (e.g. > 100,000 copies) of some DNA sequences, derivate sequences (differing in 1 or 2 bases) were frequently observed and removed together with sequences identified as spill-over from one sample to another due to sequencing errors in barcodes (see Materials and Methods as well as Supplementary Fig. S1,S2). Nearly 50% of the initial DNA sequences were discarded in the filtering process resulting in 161,072 (sd = 82,792) peptides per condition on average. To diminish the effect of amplification bias as well as bias towards samples with high number of sequencing reads, the internal rank in each sample, and not the actual sequencing count, for each peptide were used in downstream analyses.

### Examination of the phage selection process

**Figure 2:**
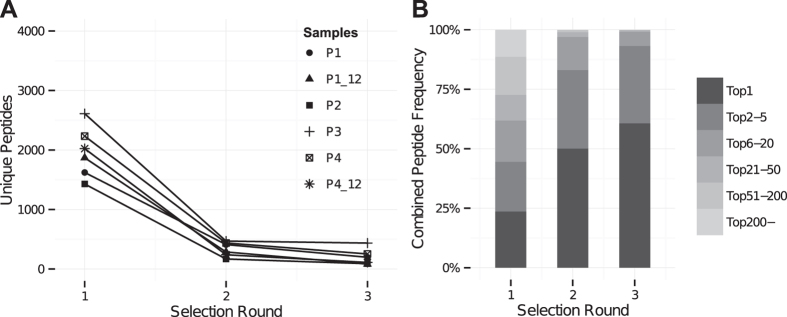
**(A)** A plot of the number of unique peptides identified in each patient sample for selection round 1–3. In the first selection round an average of 1827 (sd = 642) unique peptides were obtained, dropping to 313 (sd = 150) and finally 170 (sd = 111) unique peptides after the second and third selection round, respectively. Results were similar for control samples as shown in Supplementary Fig. S3. **(B)** A stacked bar chart showing the combined frequency of certain rank intervals. Specifically, the frequency of the most frequent peptide (Top1) along with the combined frequencies of the peptides ranked 2–5, 6–20, 21–50, 51–200 and below 200. The average of the combined frequencies for all patients is shown. Results were similar for control samples as shown in Supplementary Fig. S3.

As peptide sequences were obtained for each selection round it was possible to monitor the change in peptide frequencies over the course of successive biopanning rounds. The total number of peptide sequences varied across samples, however, no correlation with selection round was observed (Supplementary Table S2). However, the number of unique peptides in each sample decreased considerably with each subsequent selection round (Fig. 2A and Supplementary Fig. S3). For each sample an average of 89% (sd = 4%) of the peptides identified in round 2 were also present in round 1, excluding peptides with a read count of 1. This was the case for 81% (sd = 9%) of the peptides when comparing round 3 with round 2. These observations indicated that the sequencing depth was adequate to identify the majority of the selected phages.

**Figure 3:**
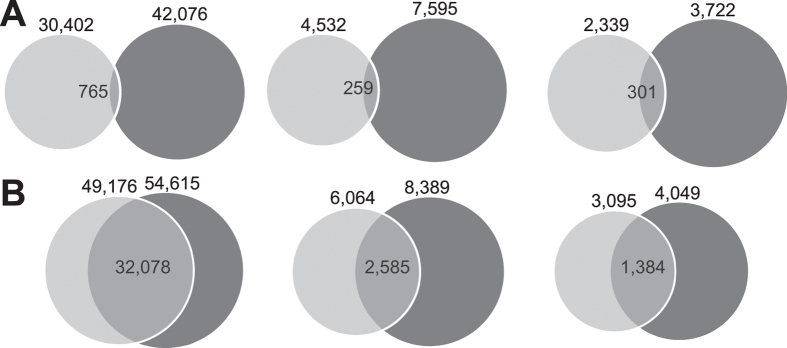
**(A)** Venn diagrams showing the overlap in identical, unique peptides between patient samples (light circle) and controls (dark circle) for each selection round. The exact numbers have been included in the figure. **(B)** Venn diagrams showing the overlap in similar peptides between patient samples (light circle) and controls (dark circle) for each selection round. Similarity was determined by pairwise alignment using an alignment threshold established as the 99.999 percentile alignment score for 10 million random peptide comparisons. The specific numbers of peptides in each group have been included in the figure.

We also observed that from round 1 to 3 a few peptides had greatly increasing frequencies and ended up dominating each sample (Fig. 2B, Table 1, Supplementary Fig. S3 and Supplementary Table S5). There was a great overlap in the most frequent peptides from round 1 to 3, e.g. for the patient samples an average of 6 (range 4–7) of the ten most frequent peptides from round 1 were also observed after round 3. However, these dominating peptides were often also observed in control samples indicating that they were TUPs with a non-specific selection advantage. In fact, on average 6 out of the 10 most frequent peptides in each selection round were also observed in the control samples (Supplementary Table S5). However, the general overlap in peptides between patient and control samples was much smaller (Fig. 3A). This indicates that the highly prevalent peptides are more likely to emerge in multiple samples, and hence be classified as TUPs. The most widespread peptide was the QLYREFN peptide, which was identified in 11 out of 12 samples. It is presented in Supplementary Table S6 alongside other TUP candidates to guide future phage display investigations.

Next, to extend the comparison of patient and control peptides beyond exact matches peptides were classified based on their similarity to other peptides. This was accomplished by pairwise alignment. The alignment threshold was established by doing 10 million random peptide comparisons, and using the 99.999 percentile alignment score. Based on this threshold, a comparison of peptide similarity between patients and controls was made (Fig. 3B). A much higher degree of overlap was observed compared to the observations based on exact matches. This indicates that peptides with sequence similarity were frequent, and that similarity had to be taken into account in further analyses.

### Identifying patient-specific peptide clusters

**Figure 4:**
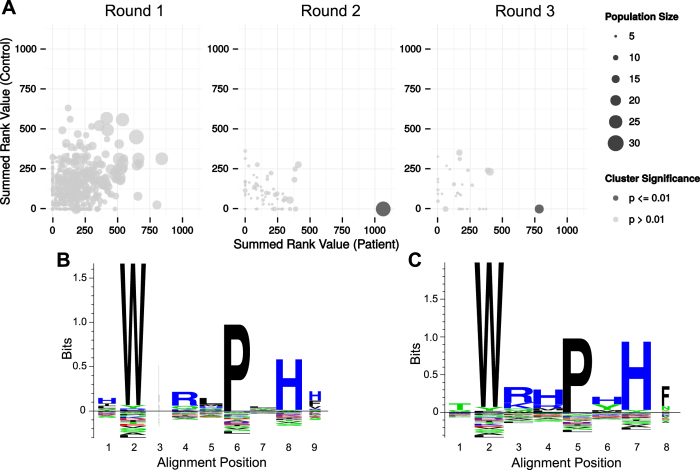
**(A)** Plots of the summed rank value for clusters obtained by pairwise-alignment and separated according to selection round (1–3). The clusters are further separated based on whether the summed rank value derives from peptides identified in patients (x-axis) or controls (y-axis). 4624, 617 and 333 clusters were identified in selection round 1, 2 and 3, respectively. One cluster in selection round 2 and 3 was significantly enriched (p-value < 0.01) for patient-specific peptides and is coloured dark. **(B)** Peptide sequence logo plot of the significant cluster obtained in round 2 and highlighted in (**A**). The logo was made by constructing position-weighted Kullback Leibler logos of the multiple alignment of the cluster peptides, using the Seq2Logo webserver^51^. Height corresponds to the amount of information contained in a specific position. Large symbols represent frequently observed amino acids. Symbols are narrow if there were many gaps in the alignment at the position. The Seq2Logo default amino acid colour coding is used (DE residues are red, NQSGTY are green, RKH are blue and the remaining are black). **(C)** Similar to (**B**) but for the significant cluster in round 3.

The next step was based on the hypothesis that selection of related peptides in multiple patients would represent key selection motifs that would be the main Ara h 1 epitope candidates. Peptides were clustered by pairwise alignment (Fig. 4). A p-value was derived by permutation of patient and control labels using a test statistic based on whether the peptides populating the cluster were derived from a patient sample or a control sample as well as their internal rank score. Accordingly, clusters that have multiple patient-specific peptides with a high rank, and where similar peptides could not be obtained in the control samples, would be provided with the lowest p-value. As such, the clustering approach takes the observed similarity between peptides from patient and control samples (Fig. 3B) into account. Interestingly, after statistical correction for the multiple testing problem (by Dunn-Bonferroni correction), a single statistically significant cluster stood out (p-value < 0.01) in selection round 2 and 3 (highlighted in Fig. 4A). In round 1 a patient-enriched cluster also had a low p-value, however, there were a large number of clusters in this round and the p-value was no longer significant after correction for multiple testing. The number of peptides in the significant cluster were 28 in selection round 2 and 17 in round 3 (Supplementary Table S7). The patient samples contributed a different number of peptides to the significant cluster (Supplementary Table S8). However, the patient sample P1 had most peptides in each of the clusters, specifically 46% and 47% of the peptides in the second and third selection round, respectively. P1, P3 and P4 contributed peptides to the significant clusters in both selection round 2 and 3. P1 and P4, which were investigated at two time points, contributed peptides at both time points. On the contrary, no peptides from P2 populated the significant clusters.

To examine which amino acid residues were the most conserved in the significant clusters, a sequence logo plot was made (Fig. 4B,C). Overall, the peptide motifs that appeared were highly similar. Closer inspection revealed that the amino acids W, P and H appeared particularly important.

### Identifying epitope candidates

**Figure 5:**
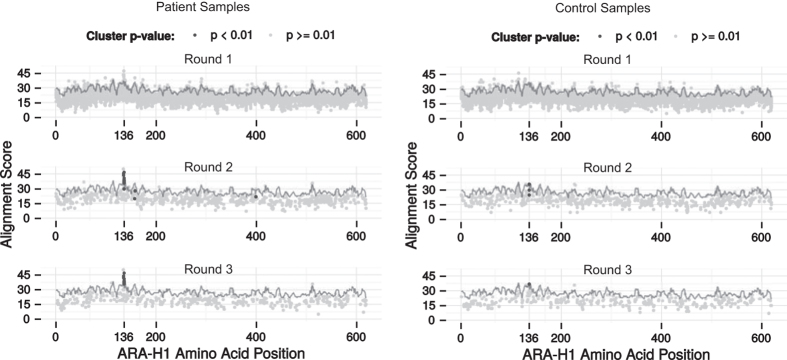
Alignment of patient-derived peptides (left) or control-derived peptides (right) to the primary sequence of Ara h 1. Peptides were mapped to Ara h 1 by aligning each peptide to overlapping 7-mers from the Ara h 1 primary sequence using pairwise alignment. Each phage peptide was plotted at the starting position of the overlapping 7-mer that provided the best alignment score. The top row is a plot for the peptides from selection round 1, while the middle and bottom row are for selection round 2 and 3, respectively. Peptides that were part of a statistically significant cluster are emphasised as dark spots. The grey line represents a significance assessment which is the 99.99 percentile alignment scores obtained after aligning 1 million random peptides to each overlapping 7-mer. Alignments with a score below zero were discarded.

In order to investigate whether the selected peptide cluster matched the primary Ara h 1 protein sequence, thus suggesting a linear epitope, all peptides were aligned to the primary sequence of Ara h 1 (Fig. 5). Multiple patient-derived peptides aligned well around position 136. This was not the case for peptides derived from control samples as well as randomly generated peptides (Fig. 5). The amino acid sequence around this position was WRRPSH. The majority of the peptides with prominent alignment scores were part of the significant cluster identified in selection round 2 and 3 in Fig. 4.

### Epitope validation on peptide arrays

**Figure 6:**
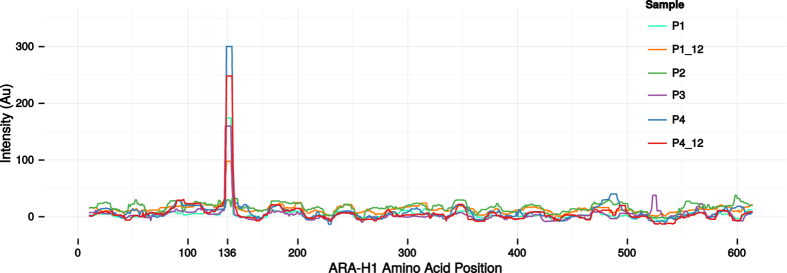
IgE reactivity of patients to Ara h 1as measured by peptide micro-arrays. The right-bound rolling median (window size 12) of the mean intensity of the triplicate 12-mer peptides overlapping each residue is shown for every patient sample. The start position of the identified epitope, position 136, has been specifically marked. For P2 no peaks were observed, whereas all the other samples showed a distinct peak at position 136.

To investigate the authenticity of the epitope suggested by alignment (Fig. 5), we investigated serum reactivity towards Ara h 1 on peptide micro-arrays. Overlapping 12-mer peptides with a single offset covering the Ara h 1 sequence were synthesised in triplicates on the chip (a total of 1845 peptides). Binding of serum IgE to the peptides was then investigated (Fig. 6). Three out of four patients (P1, P3 and P4) showed distinct peaks of IgE reactivity at position 136 in Ara h 1, which is also where the peptides from the significant cluster aligned. These three patients were the same three patients that had peptides that aligned well to Ara h 1 at position 136 (Fig. 5) and populated the significant cluster (Fig. 4A). As such, the epitope suggested by clustering and alignment was confirmed by the peptide array observations. Both time points investigated for P1 and P4 demonstrated reactivity to the same epitope thus confirming the persistence suggested by the phage peptides. For the P2 patient no IgE reactivity was observed on the peptide array, and this patient did not contribute any peptides to the significant clusters. Similarly, no IgE reactivity was observed for control subjects (Supplementary Fig. S4).

Following the confirmation of the epitope authenticity the frequency of the peptides that enabled the epitope identification were re-examined. Overall, the peptides had low frequencies, which would have made them very difficult to detect by traditional Sanger sequencing of phage clones (Supplementary Fig. S5). Assuming that 1000 phage clones were screened, e.g. by ELISA, thereby detecting peptides with a frequency above 0.1%, only 6 and 7 out of 28 and 17 peptides in the significant clusters would have been detected in selection round 2 and 3, respectively. As a consequence, the identified epitope would have been missed for P4 in all selection rounds and also for P3 in the second selection round.

## Discussion

Harnessing the power of high-throughput sequencing can improve phage display in a number of ways. First of all, the extensive data output enables the detection of rare phages in a larger population. This feature was used to detect antibody epitopes in polyclonal serum samples. It is worth noting that the IgE antibodies that target Ara h 1 only comprise a tiny fraction of the serum antibodies, given that IgE is a rare isotype compared to e.g. IgG^39^ and, further, only a fraction of the IgE consists of peanut-specific antibodies^40^. Nonetheless, Ara h 1 epitopes could be identified in the patients.

The bioinformatic pipeline presented here, which is based on clustering and contrasting with control samples, assisted the identification of a relevant peptide motif. It is worth emphasising that the clustering approach is very general, and multiple selection motifs could, in principle, be identified simultaneously. As such, the pipeline should be widely applicable in the analysis of multi-target mixtures in a variety of settings extending beyond epitope mapping to e.g. binding assays and cell surface analysis. Furthermore, with regards to epitope mapping, partly due to the high diversity of the phage library (approximately 10^9^), the approach is not limited to mapping of linear epitopes. If a highly patient-specific motif emerged, that did not match the primary sequence of the target of interest, it could be a selection motif that mimics a conformational epitope.

Another promising observation in this study, is that a patient-specific motif could already be observed in early selection rounds, supporting the notion that deep sequencing will enable phage display experiments with fewer, and ultimately, a single selection round. By performing fewer selection rounds the selection bias can be reduced which should diminish the loss of relevant sequences^16^,^31^,^32^. Hoen *et al.* have previously suggested the idea, however, they identified phages of interest based on persistence across selection rounds^32^. Similarly, other studies have identified peptides of interest based on enrichment through consequtive biopanning rounds^33^,^41^. These approaches are obviously only possible if multiple selection rounds are sequenced or, alternatively, a thorough (and expensive) sequencing of the naїve library could be sufficient. Motif identification represents an alternative approach, that could enable the identification of peptides of interest based on sequencing a single round.

Interestingly, in this study the epitope was identified without the functional testing which is commonly part of traditional phage display. The colony picking, functional testing (e.g. by ELISA) and subsequent Sanger sequencing of positive clones is a very laborius element in phage display^9^,^42^. By omitting the functional step and performing fewer selection rounds, we suggest that the throughput of phage display can be increased, as multiple complex samples can be tested in parallel.

Deep sequencing of the phage output after each selection round enabled a thorough examination of the selection process. The general trends were highly consistent across samples. With each round fewer unique peptides were obtained; decreasing from thousands after the first round to a few hundred after three rounds. This observation is in line with the expected process of affinity selection, wherein the specificity of the phage-libraries increases with additional biopanning rounds while the diversity drops^16^,^43^.

There was a great overlap among the most frequent peptides across selection rounds. However, recurring peptides with high frequencies could also be observed in control samples indicating that they were TUPs. This finding suggests that consistently prevalent peptides identified in other studies^32^,^34^ might be TUPs, which would explain why they did not match the target. Along these lines, many phage experiments have likely been discarded, or demanded laborious optimisation, because the only hits identified were irrelevant. The prevalence of TUPs was further suggested by the observation that the peptide overlap between paired samples obtained at two time points were similar to the overlap to other patient samples (for the paired samples the median Tanimoto coefficient for unique peptides was 0.6% and 0.5% for the matching pair and 0.8% and 0.4% for the overlap to the remaining patient samples for P1 and P4, respectively).

Derda *et al.* recently identified hundreds of peptides with proliferation advantages, so-called ‘parasitic’ TUP peptides, in the Ph.D.-7^TM^ library^31^. We did not observe any overlap between the peptides we obtained, and the reported parasitic TUPs. We speculate that selection criteria and experimental context (such as library type and batch) vary greatly between phage experiments and have an extensive impact on which TUPs will be selected. The high number of TUPs we observed, most of which were not previously reported, demonstrates how critical it is to filter out these sequences. As a result, we suggest including parallel control experiments to identify putative TUPs in future experimental setups. Until TUP databases^17^,^18^ have become more comprehensive, it seems to be the optimal way to take into account the distinct differences between selection schemes. It is worth noting that careful experimental design could allow parallel analysis of two or more conditions of interest, with one acting as control for the others and vice versa. This may not be feasible for all setups, but would omit the need to include a condition, that only provides information on TUPs.

Possibly due to the sample complexity a great variety of peptides were observed in the output. Selection of related peptides across patients represented key selection motifs that assisted the epitope identification. The alignment threshold used in the clustering process was determined based on simulations with randomly generated peptides. The threshold was relatively conservative and could have been varied to increase or decrease alignment stringency. A consequence of the conservative threshold is that relevant sequences may be missed. The importance of the threshold level is underlined by the observation that some patient samples had very few peptides in the significant cluster and if the alignment threshold had been slightly more restrictive, the epitope might have been overlooked. The fact that we observe peptides of high similarity that can be clustered and form motifs underlines the importance of taking similar peptides into account instead of focusing only on identical matches. Importantly, this also applies for TUPs, where excluding peptides with exact matches in control samples, such as it has previously been done^35^, may not be sufficient. The bioinformatic approach we have presented solves this issue, since TUP sequences from patients would cluster with similar peptides obtained in control samples. Consequently, even when there are no exact matches between a patient peptide and a control peptide, clustering of patient peptides with similar control peptides would result in a mixed cluster that would be disregarded due to its p-value. Alternative clustering approaches are available for the identification of peptide motifs in large-scale data, such as MUSI^44^. However, MUSI does not contrast the results with control samples and, furthermore, duplicate copies of the same sequence are removed. We suggest that the prevalence of the peptides has some correlation with the importance of the peptides and, accordingly, we employ a rank score in our analyses.

Deep sequencing of phage libraries introduced some inherent issues that had to be addressed. Highly prevalent DNA sequences gave rise to derivative sequences due to sequencing errors (Supplementary Fig. S1) as previously reported by Matochko *et al.*^31^. We further observed that, as samples were multiplexed for deep sequencing, highly prevalent DNA sequences consistently turned up with low counts in other samples on the same sequencing chip (Supplementary Fig. S2). This is presumably due to errors in sequencing of the sample barcode. It is of vital importance for later alignment and clustering that derivative sequences (both internal ‘mutations’ and cross-sample ‘spill-over’) are eliminated. Otherwise, peptides could be assigned to the wrong sample, which could hinder TUP identification, and highly prevalent peptides would give rise to clusters, that were derived from artificial sequences. The issue with derivative sequences, which is inherent to any deep sequencing study where some DNA sequences have a very high prevalence, would clearly benefit from studies dedicated to describing and solving these issues.

There was an extensive overlap in the epitope mapping results for individual samples from phage display compared to peptide micro-arrays. Specifically, the samples that had peptides mimicking the Ara h 1 epitope were also the samples where this epitope could be identified on the peptide micro-array. This suggests that both techniques generate reproducible results of considerable accuracy. Interestingly, the identified epitope appeared to be persistent, since it could be identified by phage display as well as peptide micro-arrays in samples obtained with a 9 year interval. Of note, the identified epitope is included in a previously described epitope hotspot of Ara h 1^36^,^45^. A previous study, based on conventional phage display, further suggested conformational Ara h 1 epitopes^46^, however, no such motifs were identified in the present study. For patient P2 no epitopes were identified by phage display or peptide micro-arrays. This patient had a low level of specific IgE and we speculate that the methods were not sensitive enough to identify any epitopes or that the sample quality was low.

The coupling of phage display with high-throughput sequencing offers several advances to conventional phage display. First of all, the large data output provides an opportunity to perform in-depth studies of the phage selection process and also permits the identification of rare phages in a large population. This should improve knowledge of the parameters that affect phage selection and enable the study of multi-target samples. Here, a general bioinformatic approach has been presented, which identifies selection motifs of interest. The observed prevalence of TUPs demonstrated the necessity of examining parallel control samples, which were used as a contrast in the clustering approach. Relevant selection motifs could be identified in early selection rounds and without laborious functional screening. The increased throughput combined with the in-depth data output holds the promise to expand the use of phage display to new applications.

## Methods

### Peanut allergic patient samples

Sera from 4 peanut allergic adults (P1-4) and 4 peanut-tolerant control subjects (C1-4) were analysed alongside plasma from 4 of these individuals (P1_12, P4_12, C2p and C3p). The 2 patient plasma samples (P1_12 and P4_12) were obtained about 9 years after the patient sera samples. The 4 patients had a convincing history of peanut-related anaphylaxis according to WAO guidelines^47^. All samples were tested for peanut extract-specific IgE by ImmunoCAP and Ara h 1-specific IgE by ELISA. No Ara h 1-IgE reactivity was observed for the control samples. The protocol was approved by the local Ethical Committee of the Medical University of Vienna and written informed consent was obtained (protocol no. EK 428/2008). The samples were treated in accordance with the approved guidelines.

### Selection of Ara h 1 mimicking peptides

Figure 1 provides an overview of the selection process. In brief, 12 × 200 μL (6 mg) of M-280 tosylactivated Dynabeads (Invitrogen) were coated with 10 μg polyclonal rabbit anti-human IgE (DAKO, A0094) for 48 h at 37 °C, and blocked with 0.5% skimmed milk powder (SMP) in Phosphate Buffer Saline (PBS). Patient serum (500 μL) or plasma (1,300 μL) was diluted in PBS with 0.5% SMP and 0.05% Tween 20, and incubated overnight at 4 °C with 200 μL of the coated bead suspension. After extensive washing, 50 μL of the coated beads were used immediately for the first round of selection, while the remaining 150 μL were stored for later selection rounds.

In the first selection round 50 μL beads with immobilized IgE were blocked in 1 mL of 2% SMP in PBS for 1 h at room temperature and washed extensively. The beads were then incubated overnight at 4 °C with 10 μL (~2 × 10^11^) phages from a library of phages displaying random heptamer peptides (Ph.D.-7^TM^, New England BioLabs). This was followed by extensive washing and negative selection; specifically 1 h incubation in 10 mL PBS with 0.5% SMP and 0.05% Tween 20 and 8 washing steps each consisting of 10 min incubation in 10 mL PBS with 0.05% Tween 20. Next, phages of interest were eluted by adding 25 μg Ara h 1 protein. The Ara h 1 was purified as previously described by Bøgh *et al.*^48^. The purity of Ara h 1 was confirmed by reverse phase high-performance liquid chromatography to be >99%. Size exclusion chromatography demonstrated that the Ara h 1 was present as a trimer. Eluted phages were amplified by direct infection of ER2738 Escherichia coli cells (New England Biolabs). Phage amplification was done at 37 °C for 4.5 hours. Amplified phages were precipitated using PEG/NaCl and titered. The second and third selection rounds were carried out in an identical way, using ~2 × 10^11^ of the phages selected in the previous round.

### High-throughput Sequencing

Amplified phage eluates from each of the 3 selection rounds were subjected to PCR using the Phusion High-Fidelity DNA polymerase (New England Biolabs). To mitigate PCR bias, the PCR reactions were carried out in triplicates and the number of cycles was limited to 25. The PCR was carried out as recommended by the manufacturer, using 25 μL reactions with 1 μL 1:10 diluted phage eluates as template and an annealing temperature of 55 °C. The variable part of the phage library was specifically amplified using barcoded primers (Supplementary Table S1). The corresponding PCR fragment was extracted from a 2% agarose gel using the Qiaquick Gel Extraction Kit (Qiagen). The triplicate PCR reactions were pooled prior to the gel extractions. The DNA concentration was determined using a Qubit fluorometer (Life) and the fragment size was confirmed using a 2100 Bioanalyzer (Agilent) following manufacturer’s protocol.

Next, sequencing adaptors were ligated onto the DNA fragments using the Ion Plus fragment library kit (Life), and the fragment size and concentration was confirmed using a Bioanalyzer (Agilent) and Qubit fluorometer (Life), respectively. Subsequently, the DNA fragments were attached to Ion Sphere Particles (ISPs) and amplified by emulsion PCR according to the Ion OneTouch 200 protocol. The ISPs were loaded onto an Ion 316 chip and sequenced on an Ion Torrent PGM using the Ion Torrent sequencing kit 200 v2, as described by the manufacturer.

### Converting sequencing reads to peptides and quality filtering

DNA reads obtained from the sequencing was first filtered by sample barcodes. Reads that did not match any of the barcodes, both forward and reverse complement, were removed. The DNA reads were then translated in all six reading frames and aligned to the protein regions of the phage protein that flank the displayed 7-mer peptide using the BLAST algorithm^49^. Then 7-mer peptides and their originating DNA sequences were extracted from reads that produced meaningful alignments; i.e. the protein sequence aligned to both flanking regions with a 7-mer peptide gap.

The peptides were further processed by i) removing peptides where at least 1 DNA base pair (bp) coding for the peptide were assigned a Phred quality score below 20 and ii) removing reads where the DNA sequence did not match the NNK-codon pattern intrinsic to the Ph.D.-7^TM^ phage library (N = any nucleotide, K = G or T).

Some sequences had a very high prevalence within a sample. A consequence of high-throughput sequencing read errors^19^,^31^ could be that such abundant sequences might artificially give rise to similar sequences with a low abundance, differing from the abundant sequence at 1 or 2 positions. In fact, a linear relationship was observed between the prevalence of abundant sequences and the prevalence of similar “derivative” sequences (Supplementary Fig. S1). This suggests that the derivative peptides are indeed read error variations of the abundant peptide. As a result, sequences that were at least 500 or 10,000 times less prevalent than a similar (high abundant) sequence in the same sample, differing by just one or two base pairs, respectively, were removed. These conservative thresholds were established to ensure the relevance of the detected peptides. Furthermore, when two subjects were analysed on the same Ion Torrent chip, highly abundant sequences in one patient were sometimes observed with a low prevalence in the other subject. The relationship in prevalence was, once again, linear and such relationship could not be observed across different sequencing chips (Supplementary Fig. S2). The derivative sequences likely originate from sequencing errors in the barcode sequence leading to a mislabelling of the sample. To avoid this issue, peptides of low abundance were removed if they were also observed with at least 100 times higher prevalence in the other subject tested on the same Ion Torrent chip. Finally, the obtained peptides were compared to a list of “parasitic” phages with a suspected proliferation advantage^31^, however, no overlap was observed and no peptides were removed. An overview of the number of reads that passed each step of quality filtering can be found in Supplementary Table S2-4.

### Peptide clustering

To remove bias towards samples with a higher number of sequencing reads, each unique peptide in a sample was assigned a normalised rank score based on the following formula:


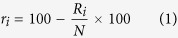


Here *R*_*i*_ is the rank of peptide *i* within a specific sample. Peptides with the same prevalence were given an average rank score. As a result of the above conversion, the most prevalent peptide in each sample will be given a rank score of 100, and the least prevalent peptide will have a rank score of 0. The rank values were summed across samples and used for sorting peptides prior to clustering.

Peptides of each sample were clustered according to the Hobohm I algorithm using pairwise alignment as distance function^50^ and an alignment score threshold of 31. The alignment threshold of 31 was established as the 99.999 percentile of 10 million pair-wise alignments of random peptides, using a standard Smith-Waterman alignment algorithm (using BLOSUM50, gap-opening and extension penalties of 5 and 1, respectively, and a minimum alignment length of 6). This alignment threshold was also used for an overall comparison of the peptides in patient and control samples.

To establish patient-related significance each cluster was analysed by patient-control contrasting. The difference, *t*, between summed rank-scores, *r*_*i*_, assigned to patients and controls within a single cluster were calculated and used as test-statistic. The null-hypothesis assumes that the test statistic would be equal or higher to that of random clusters established by permutation, i.e. shuffling the patient-control labels 10,000 times. A p-value of the observed test-statistic was calculated as the number of random clusters having a lower test-statistic divided by the number of permutations (10,000), as shown in Equation 2:


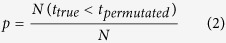


The null-hypothesis was rejected for clusters with a p-value lower than 0.01/*n* where *n* is the number of clusters formed. Hence, such clusters had a significant patient-control contrast and were assumed to be obtained from allergen-specific selection.

### Representing significant clusters by sequence motifs

Cluster motif visualisation was done by constructing position-weighted Kullback Leibler logos of the multiple alignment of the cluster peptides, using the Seq2Logo webserver^51^. The multiple alignment was performed using a MAFFT-incorporating Needleman-Wunsch global pairwise alignment algorithm using 1,000 cycles of iterative refinement for increased accuracy.

### Aligning peptides to the Ara h 1 sequence

Peptides that were selected from the phage libraries were mapped to Ara h 1 by aligning each peptide to overlapping 7-mers from the Ara h 1 primary sequence (Uni-prot ID: P43238) using the same alignment parameters as for the peptide clustering. Each phage peptide was assigned the starting position of the overlapping 7-mer that provided the best alignment score. Alignments with a score below zero were discarded. The significance of alignment scores was assessed by aligning 1 million random peptides to each overlapping 7-mer and reporting the 99.99 percentile of the alignment scores.

### High-density peptide micro-arrays

A peptide library was generated *in silico* for synthesis on high-density peptide micro-arrays. The library consisted of single-offset overlapping 12-mer peptides covering the primary sequence of Ara h 1 (Uni-prot ID: P43238) in triplicates (1845 peptides). Peptide synthesis was accomplished by light-directed array synthesis in a Roche-NimbleGen Maskless Array Synthesizer (MAS) using an amino functionalised substrate coupled with 6-amino hexanoic acid as a spacer and amino acid derivatives carrying a photosensitive 2-(2-Nitrophenyl) propyl-oxy-carbonyl-group (NPPOC). Coupling of amino acids was done using pre-activated amino acid with activator (HOBT/HBTU) and Ethyl-di-iso-propylamine in DMF for 5–7 minutes before flushing the substrate. Cycles of coupling were repeated until 12-mer peptides were synthesized. Intermediate washes on the arrays were done with N-Methyl-2-pyrrolidone (NMP) and site-specific cleavage of the NPPOC group was accomplished by irradiation of an image created by a Digital Micro-Mirror Device (Texas Instruments, SXGA + graphics format), projecting light with a 365 nm wavelength. Final de-protection to cleave off the side-chain protecting groups of the amino acids was done with Trifluoroacetic acid(TFA)/Water/Triisopropylsilane for 30 minutes. The final micro-arrays were incubated overnight at 4 °C with individual sera mixed with binding buffer in a 1:5 dilution in a final volume of 20 μL. This was followed by incubation with goat anti-Human IgE conjugated with tetramethylrhodamine (TRITC) (Life, A18798) at room temperature for 3 hours. Serum from P1 and P3 were also evaluated in a 1:2 dilutions with binding buffer. Finally, the arrays were scanned using a MS200 microarray scanner, and signals were extracted using NimbleGen DEVA signal extraction software.

## Additional Information

**How to cite this article**: Christiansen, A. *et al.* High-throughput sequencing enhanced phage display enables the identification of patient-specific epitope motifs in serum. *Sci. Rep.* **5**, 12913; doi: 10.1038/srep12913 (2015).

## Supplementary Material

Supplementary Information

## Figures and Tables

**Table 1:**
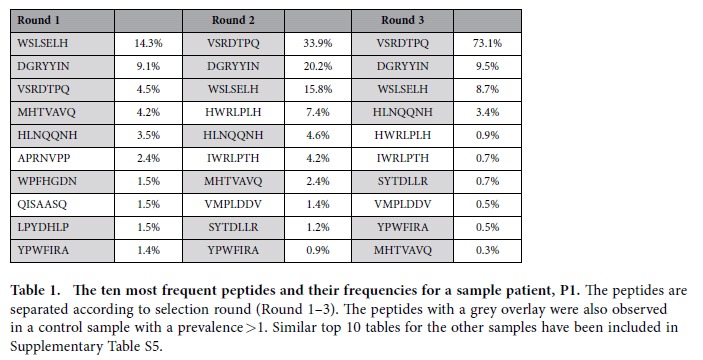
The ten most frequent peptides and their frequencies for a sample patient, P1.

The peptides are separated according to selection round (Round 1–3). The peptides with a grey overlay were also observed in a control sample with a prevalence >1. Similar top 10 tables for the other samples have been included in Supplementary Table S5.
