# Supplementary material for: High-throughput sequencing enhanced phage display enables the identification of patient-specific epitope motifs in serum
Source: Sci Rep. 2015 Aug 6;5:12913. doi: 10.1038/srep12913 (PMC4650709; doi:10.1038/srep12913)
Supplement: Supplementary Information [file srep12913-s1.pdf]

Supplementary information - "High-throughput sequencing enhanced phage display enables the identification of patient-specific epitope motifs in serum"

Anders Christiansen[1], Jens V. Kringelum[2], Christian S. Hansen[2], Katrine L. Bøgh[3], Eric Sullivan[4], Jigar Patel[4], Neil M. Rigby[5], Thomas Eiwegger[6], Zsolt Szépfalusi[6], Federico de Masi[2], Morten Nielsen[2,7], Ole Lund[2], Martin Dufva[1]*

[1] Department of Micro- and Nanotechnology, Technical University of Denmark, Kgs. Lyngby, Denmark,

[2] Center for Biological Sequence Analysis, Technical University of Denmark, Kgs. Lyngby, Denmark

[3] National Food Institute, Technical University of Denmark, Søborg, Denmark

[4] Roche NimbleGen, Madison, Wisconsin, the United States of America

[5] Institute of Food Research, Norwich, United Kingdom

[6] Department of Paediatrics, Medical University of Vienna, Vienna, Austria

[7] Instituto de Investigaciones Biotecnológicas, Universidad Nacional de San Martín, Buenos Aires, Argentina

* Correspondence and requests for materials should be addressed to M.D.

(martin.dufva@nanotech.dtu.dk)

5´-*AAGAGGATTC* GAT TTC GCA ATT CCT TTA GTG
5´-*CTGCAAGTTC* GAT TTC GCA ATT CCT TTA GTG
5´-*TCTAACGGAC* GAT TTC GCA ATT CCT TTA GTG
5´-*TTGGAGTGTC* GAT TTC GCA ATT CCT TTA GTG
5´-*TCTGGATGAC* GAT TTC GCA ATT CCT TTA GTG
5´-*TCTATTCGTC* GAT TTC GCA ATT CCT TTA GTG
Reverse: 5´-GCT AAA CAA CTT TCA ACA G

Supplementary Table S1. Primer and barcode DNA sequences used for PCR. The barcode element has been italicised. The reverse primer in the bottom row was used for all reactions.

Sequencing Run	Patient	Selection round	#reads w barcode	Peptides called	NNK filtering	Quality filtering
**1**	C2P	1	270337	199212 (73%)	198283 (73%)	151466 (56%)
**1**	C2P	2	348124	179438 (51%)	177502 (50%)	93727 (26%)
**1**	C2P	3	341409	152896 (44%)	150871 (44%)	65241 (19%)
**1**	P4_12	1	337208	193715 (57%)	191271 (56%)	123682 (36%)
**1**	P4_12	2	267119	161249 (60%)	160271 (59%)	110706 (41%)
**1**	P4_12	3	317513	216935 (68%)	215009 (67%)	141279 (44%)
**2**	C1	1	372884	320282 (85%)	318595 (85%)	209704 (56%)
**2**	C1	2	247717	201535 (81%)	200532 (80%)	141377 (57%)
**2**	C1	3	229598	188859 (82%)	188057 (81%)	110312 (48%)
**2**	C3	1	493126	433610 (87%)	429495 (87%)	312966 (63%)
**2**	C3	2	315301	275409 (87%)	274312 (87%)	218529 (69%)
**2**	C3	3	444588	398677 (89%)	397525 (89%)	352758 (79%)
**3**	C2	1	187617	145280 (77%)	144427 (76%)	87672 (46%)
**3**	C2	2	163062	139999 (85%)	139370 (85%)	92461 (56%)
**3**	C2	3	191137	165666 (86%)	164854 (86%)	110574 (57%)
**3**	C4	1	314801	264167 (83%)	260828 (82%)	181312 (57%)
**3**	C4	2	150530	111450 (74%)	110413 (73%)	73886 (49%)
**3**	C4	3	211778	160403 (75%)	158561 (74%)	96523 (45%)
**4**	P2	1	389423	336846 (86%)	334691 (85%)	225844 (57%)
**4**	P2	2	248397	222564 (89%)	221625 (89%)	178082 (71%)
**4**	P2	3	269238	239172 (88%)	238660 (88%)	198232 (73%)
**4**	P4	1	470407	373218 (79%)	370282 (78%)	247124 (52%)
**4**	P4	2	277800	223134 (80%)	222006 (79%)	149424 (53%)
**4**	P4	3	460451	395964 (85%)	392917 (85%)	278072 (60%)
**5**	P1_12	1	259866	148832 (57%)	146965 (56%)	102337 (39%)
**5**	P1_12	2	255146	150404 (58%)	148385 (58%)	111000 (43%)
**5**	P1_12	3	247601	135410 (54%)	134204 (54%)	99317 (40%)
**5**	C3P	1	301990	173918 (57%)	172066 (56%)	111241 (36%)
**5**	C3P	2	234836	103078 (43%)	102275 (43%)	57059 (24%)
**5**	C3P	3	246973	99754 (40%)	98743 (39%)	70327 (28%)
**6**	P3	1	573487	459349 (80%)	456976 (79%)	304519 (53%)
**6**	P3	2	277907	201369 (72%)	200826 (72%)	126143 (45%)
**6**	P3	3	168015	94123 (56%)	93657 (55%)	65886 (39%)
**6**	P1	1	499211	425951 (85%)	424085 (84%)	307978 (61%)
**6**	P1	2	349713	317818 (90%)	316771 (90%)	256409 (73%)
**6**	P1	3	362151	329429 (90%)	328668 (90%)	262470 (72%)

Supplementary Table S2. Number of sequences in each step of the processing. The provided numbers are the number of sequences that remains at the particular stage. The percentage denotes the percentage of remaining sequences compared to the initial assignment based on barcodes. In brief the steps were (i) associating sequences with a sample based on barcode, (ii) calling 7-mer peptides and (iii) filtering peptides based NNK codon pattern and (iv) quality filtering. The steps are covered in greater detail in the Materials and Methods section.

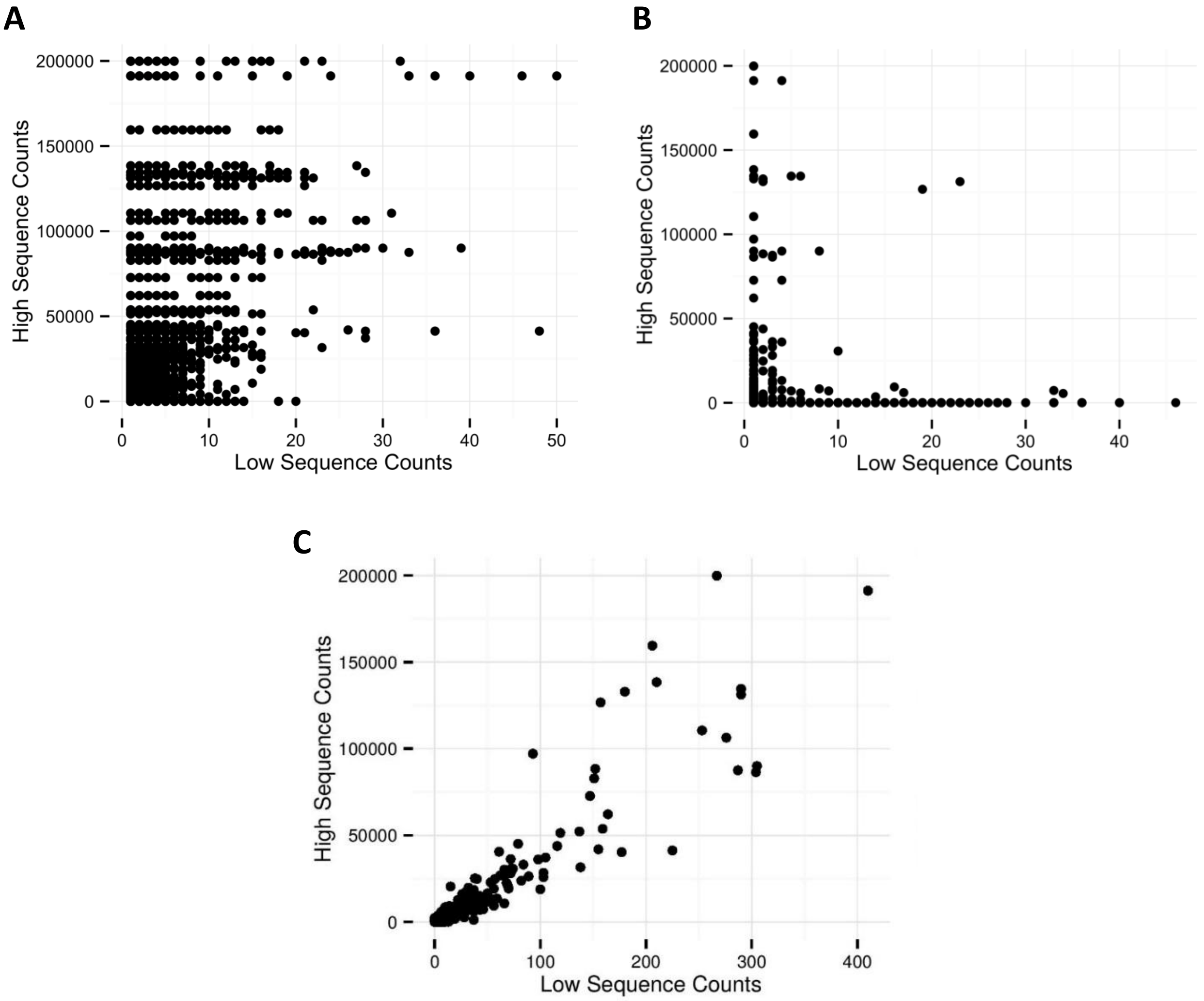

Supplementary Figure S1. (A) Derivative sequences due to high-throughput sequencing errors. The relationship between the prevalence of f a high-frequent peptide (“High Sequence Counts”) and the prevalence of similar peptides where the DNA sequence varies at a single position (“Low Sequence Counts”) has been plotted. Note that the axes have different scales. The horizontal rows of observations that are visible at high prevalences corresponds to the various derivative sequences that are observed from a single high-frequent peptide. **(B)** As (A) but plotting the prevalence of derivative sequences where there are two deviations in the DNA sequences compared to the high-frequent peptide. **(C)** As (A) but depicting the sum of all derivative sequences on the “Low Sequence Counts” axis for each high-frequent peptide. A linear relationship between the prevalence of the high-frequent peptide and the sum of the derivative sequences is observed, where peptides with higher prevalences give rise to more derivative sequences. Such a relation is to be expected if the derivative sequences are due to sequencing errors.

Threshold details: The applied thresholds of 500 for single-base deviations (shown in A) and 10,000 for double-base deviations (shown in B) where empically determined. Taking the 500-fold threshold as an example, sequences were removed if they were at least 500 times less prevalent than a similar sequence (differing by a single base pair) in the same sample. Translating this to a per-base error rate was done by the formula P^{20} × ((1-P) / 3) = 1/500 × P^21, where P is the per-base accuracy and 1-P is the per-base error rate. (1-P) /3 assumes that the change to any of the three other bases are equally distributed. Solving for P yields P = 0.994, thus the 500-fold threshold corresponds to a per-base sequencing error rate of 0.6%.

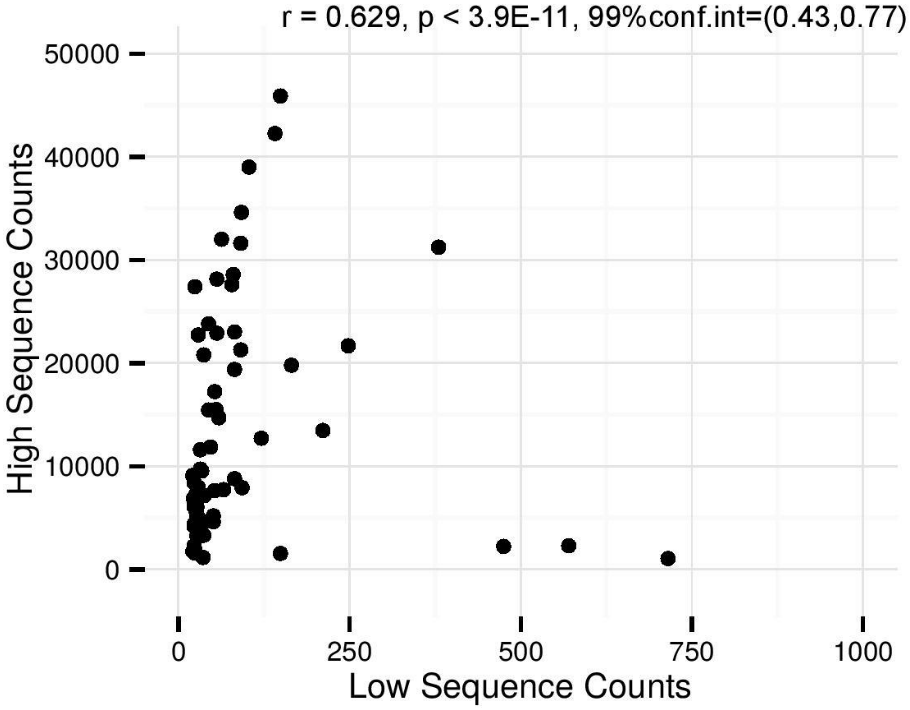

Supplementary Figure S2. Derivative sequences due to high-throughput sequencing errors give rise to "spill-over" between samples. The relationship between the prevalence of a high-frequent peptide ("Count High") and the prevalence of the same peptides in another subject ("Count Low"), analysed on the same sequencing chip has been plotted. Note that the axes have different scales. A maximum of 50,000 and 1,000 is plotted for "Count High" and "Count Low", respectively, has been plotted. The summed prevalence for each peptide across the three selection rounds is depicted. A Pearson correlation coefficient has been calculated and included.

Sequencing Run	Patient	Selection round	Peptide sequences	"Derivative" filtering	"Spill-over" filtering
**1**	C2P	1	151466	151025 (99%)	150936 (99%)
**1**	C2P	2	93727	93495 (99%)	93489 (99%)
**1**	C2P	3	65241	65010 (99%)	64979 (99%)
**1**	P4_12	1	123682	123432 (99%)	123378 (99%)
**1**	P4_12	2	110706	110385 (99%)	110380 (99%)
**1**	P4_12	3	141279	140905 (99%)	140880 (99%)
**2**	C1	1	209704	209320 (99%)	208751 (99%)
**2**	C1	2	141377	141003 (99%)	140477 (99%)
**2**	C1	3	110312	110128 (99%)	109509 (99%)
**2**	C3	1	312966	312506 (99%)	311830 (99%)
**2**	C3	2	218529	218051 (99%)	217719 (99%)
**2**	C3	3	352758	352267 (99%)	351732 (99%)
**3**	C2	1	87672	87501 (99%)	87321 (99%)
**3**	C2	2	92461	92260 (99%)	92071 (99%)
**3**	C2	3	110574	110383 (99%)	110095 (99%)
**3**	C4	1	181312	181039 (99%)	180491 (99%)
**3**	C4	2	73886	73665 (99%)	73481 (99%)
**3**	C4	3	96523	96349 (99%)	96013 (99%)
**4**	P2	1	225844	225377 (99%)	224941 (99%)
**4**	P2	2	178082	177633 (99%)	177381 (99%)
**4**	P2	3	198232	197940 (99%)	197718 (99%)
**4**	P4	1	247124	246787 (99%)	245951 (99%)
**4**	P4	2	149424	148951 (99%)	148441 (99%)
**4**	P4	3	278072	277535 (99%)	276623 (99%)
**5**	P1_12	1	102337	102163 (99%)	102149 (99%)
**5**	P1_12	2	111000	110699 (99%)	110692 (99%)
**5**	P1_12	3	99317	98988 (99%)	98969 (99%)
**5**	C3P	1	111241	110945 (99%)	110894 (99%)
**5**	C3P	2	57059	56864 (99%)	56836 (99%)
**5**	C3P	3	70327	70080 (99%)	70048 (99%)
**6**	P3	1	304519	303877 (99%)	302501 (99%)
**6**	P3	2	126143	125726 (99%)	124944 (99%)
**6**	P3	3	65886	65616 (99%)	63626 (96%)
**6**	P1	1	307978	307130 (99%)	306615 (99%)
**6**	P1	2	256409	255457 (99%)	255134 (99%)
**6**	P1	3	262470	261885 (99%)	261614 (99%)

Supplementary Table S3. Number of sequences filtered due to high-throughput sequencing errors. "Derivative" filtering comprise the removal of the derivative sequences plotted in Supplementary Figure S1, i.e. peptides in a sample that are very similar to a high-frequent peptide in the same sample. "Spill-over" filtering encompasses the removal of the derivative sequences plotted in Supplementary Figure S2, i.e. peptides in a sample that are very similar to a high-frequent peptide in a different subject, analysed on the same sequencing chip. The provided numbers are the number of peptide sequences that remains at the particular stage. The percentage denotes the percentage of remaining peptides after the specific filtering step.

Sequencing Run	Patient	Selection round	Unique Peptides	"Derivative" filtering	"Spill-over" filtering
**1**	C2P	**1**	1655	1552 (93%)	1531 (92%)
**1**	C2P	2	262	166 (63%)	163 (62%)
**1**	C2P	3	147	81 (55%)	70 (47%)
**1**	P4_12	1	2181	2033 (93%)	2023 (92%)
**1**	P4_12	2	362	242 (66%)	239 (66%)
**1**	P4_12	3	206	119 (57%)	110 (53%)
**2**	C1	1	1839	1582 (86%)	1519 (82%)
**2**	C1	2	456	358 (78%)	285 (62%)
**2**	C1	3	250	208 (83%)	150 (60%)
**2**	C3	1	2045	1846 (90%)	1770 (86%)
**2**	C3	2	811	625 (77%)	555 (68%)
**2**	C3	3	474	365 (77%)	291 (61%)
**3**	C2	1	799	696 (87%)	654 (81%)
**3**	C2	2	228	165 (72%)	117 (51%)
**3**	C2	3	203	156 (76%)	97 (47%)
**3**	C4	1	3367	3210 (95%)	3182 (94%)
**3**	C4	2	637	503 (78%)	481 (75%)
**3**	C4	3	307	205 (66%)	184 (59%)
**4**	P2	1	1660	1529 (92%)	1429 (86%)
**4**	P2	2	349	227 (65%)	168 (48%)
**4**	P2	3	218	145 (66%)	89 (40%)
**4**	P4	1	2484	2279 (91%)	2233 (89%)
**4**	P4	2	614	459 (74%)	437 (71%)
**4**	P4	3	419	293 (69%)	253 (60%)
**5**	P1_12	1	1974	1875 (94%)	1869 (94%)
**5**	P1_12	2	436	292 (66%)	286 (65%)
**5**	P1_12	3	203	88 (43%)	82 (40%)
**5**	C3P	1	1681	1504 (89%)	1488 (88%)
**5**	C3P	2	260	167 (64%)	157 (60%)
**5**	C3P	3	186	88 (47%)	76 (40%)
**6**	P3	1	3078	2711 (88%)	2611 (84%)
**6**	P3	2	643	550 (85%)	469 (72%)
**6**	P3	3	594	540 (90%)	436 (73%)
**6**	P1	1	2255	1771 (78%)	1622 (71%)
**6**	P1	2	754	522 (69%)	410 (54%)
**6**	P1	3	390	278 (71%)	197 (50%)

Supplementary Table S4. Number of unique peptide sequences filtered due to high-throughput sequencing errors. The table is similar to Supplementary Table S2, however, the number of unique peptides is given instead of total peptide sequence numbers. "Derivative" filtering comprise the removal of the derivative sequences plotted in Supplementary Figure S1, i.e. peptides in a sample that are very similar to a high-frequent peptide in the same sample. "Spill-over" filtering encompasses the removal of the derivative sequences plotted in Supplementary Figure S2, i.e. peptides in a sample that are very similar to a high-frequent peptide in a different subject, analysed on the same sequencing chip. The percentage denotes the percentage of remaining unique peptides after the specific filtering step.

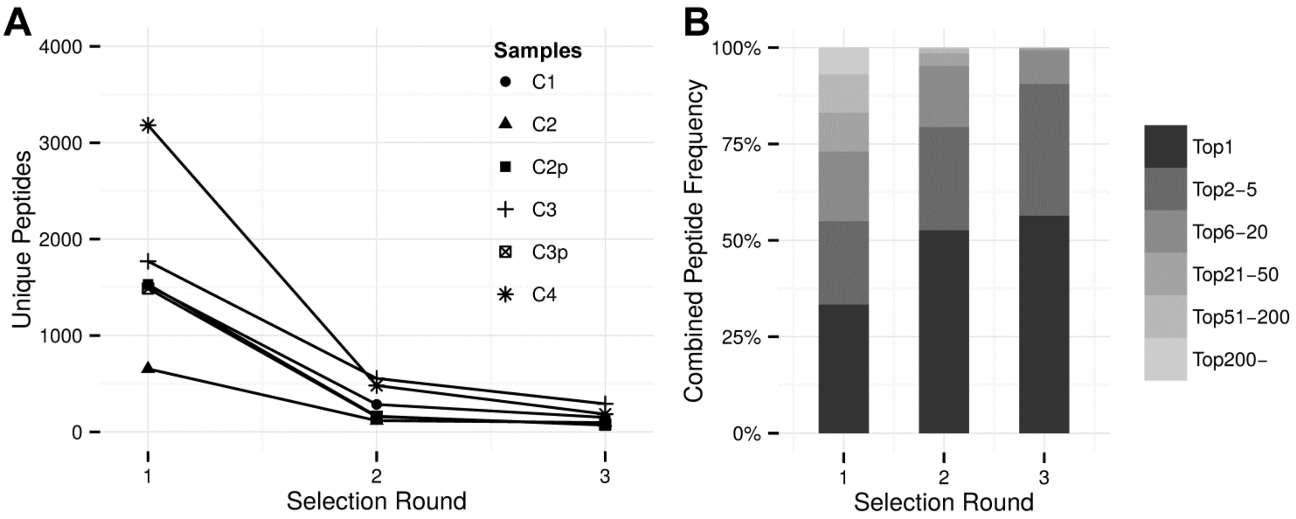

Supplementary Figure S3. The selection process in control samples. **(A)** A plot of the number of unique peptides identified in each control sample for selection round 1-3. **(B)** A stacked bar chart showing the combined frequency of certain rank intervals. Specifically, the frequency of the most frequent peptide (Top1) along with the combined frequencies of the peptides ranked 2-5, 6-20, 21-50, 51-200 and below 200, based on their frequency in a sample. The average of the combined frequencies for all control samples is shown.

PATIENT: P1					
WSLSELH	14.3%	VSRDTPQ	33.9%	VSRDTPQ	73.1%
DGRYYIN	9.1%	DGRYYIN	20.2%	DGRYYIN	9.5%
VSRDTPQ	4.5%	WSLSELH	15.8%	WSLSELH	8.7%
MHTVAVQ	4.2%	HWRLPLH	7.4%	HLNQQNH	3.4%
HLNQQNH	3.5%	HLNQQNH	4.6%	HWRLPLH	0.9%
APRNVPP	2.4%	IWRLPTH	4.2%	IWRLPTH	0.7%
WPFHGDN	1.5%	MHTVAVQ	2.4%	SYTDLLR	0.7%
QISAASQ	1.5%	VMPLDDV	1.4%	VMPLDDV	0.5%
LPYDHLP	1.5%	SYTDLLR	1.2%	YPWFIRA	0.5%
YPWFIRA	1.4%	YPWFIRA	0.9%	MHTVAVQ	0.3%

PATIENT: P2					
DNYDSTA	59.8%	DNYDSTA	74.0%	DNYDSTA	64.1%
MLHELLE	3.0%	VSGLLVE	7.3%	ENHVHVR	15.5%
RVASLAP	2.7%	ENHVHVR	5.3%	VSGLLVE	12.7%
ENHVHVR	2.6%	MLHELLE	4.3%	YPWFIRA	4.7%
NYFKHEA	2.3%	NSPELTS	2.1%	MLHELLE	2.5%
DAIPTSV	1.8%	DAIPTSV	1.7%	SLLGQTP	0.1%
VSGLLVE	1.8%	SLLGQTP	1.1%	NSPELTS	0.1%
ELRNENT	1.8%	YPWFIRA	0.7%	SPTTTYD	0.1%
NSPELTS	1.1%	NYFKHEA	0.5%	TALRTIS	0.1%
NGHSIHT	1.0%	ISPLSVP	0.5%	ISPLSVP	0.0%

PATIENT: P3					
EVMGRLA	10.4%	EVMGRLA	72.1%	EVMGRLA	64.9%
STTPHSR	7.5%	MVPPSYD	5.8%	MVPPSYD	8.7%
MVPPSYD	3.4%	QLIHWRH	4.8%	VKFDKYV	8.5%
SSIGGQD	2.9%	QLYREFN	3.6%	AMSHSKQ	5.4%
QLYREFN	2.8%	SSNQFHQ	1.5%	QLIHWRH	3.1%
YLGFDVH	1.8%	HSILSNL	1.5%	YLGFDVH	1.2%
SQNTKYI	1.5%	YLGFDVH	1.4%	WSLSELH	1.1%
QLIHWRH	1.3%	AQYVAVG	0.9%	HSILSNL	0.9%
MVQHYAE	1.2%	SSIGGQD	0.8%	LKFDTPI	0.9%
HSILSNL	1.2%	ATDEIVP	0.8%	QLYREFN	0.7%

PATIENT: P4					
SLLGQTP	16.5%	SLLGQTP	41.9%	SLLGQTP	50.0%
STIVAEM	6.8%	SAAWNKS	16.0%	SAAWNKS	18.9%
GFTFQPS	5.3%	STIVAEM	12.9%	STIVAEM	13.1%
SAAWNKS	4.7%	GTGSQAS	6.6%	GTGSQAS	6.2%
TNLSHVP	3.4%	GFTFQPS	5.9%	QTWLEMG	4.4%
QTWLEMG	3.3%	QTWLEMG	4.9%	GFTFQPS	2.5%
LLADMHA	2.6%	TNLSHVP	2.3%	KGIYHQV	0.8%
GTGSQAS	2.0%	SIIARPG	1.8%	STPATLI	0.7%
LVYVDMH	1.0%	STPATLI	1.2%	SLSSAWW	0.6%
ISYLSVT	0.9%	LLADMHA	0.8%	TNLSHVP	0.5%

PATIENT: P1_12					
AMSPLNK	16.2%	MPTWLHH	29.9%	GASESYL	36.4%
MPTWLHH	13.8%	AMSPLNK	17.5%	MPTWLHH	26.1%
NVRLPYQ	9.2%	GASESYL	10.4%	IENRIYR	13.4%
NLLDSLH	6.0%	GVFISYN	5.3%	AMSPLNK	8.6%
NPTHPIY	3.5%	FASRSDT	4.8%	FASRSDT	3.6%
FASRSDT	2.7%	IENRIYR	4.7%	NPPWFHT	2.2%
IENRIYR	2.3%	NVRLPYQ	3.5%	GVFISYN	2.2%
GVFISYN	2.2%	IPNGHFT	3.4%	IPNGHFT	1.4%
LSKINSP	2.1%	NLLDSLH	3.3%	NVRLPYQ	0.7%
GNEVMTY	1.6%	NPTHPIY	2.4%	NLLDSLH	0.7%

PATIENT: P4_12					
LSANHWV	24.5%	VPNIVTQ	48.7%	VPNIVTQ	75.5%
VPNIVTQ	7.5%	LSANHWV	24.3%	LSANHWV	5.6%
VTRDSNH	7.2%	SFNLPNT	5.1%	HGGVRLY	5.0%
SHPLWNS	5.6%	GMMSSPP	4.7%	SFNLPNT	3.4%
SFNLPNT	4.1%	YVTRTPY	1.8%	GMMSSPP	3.1%
LPFINSD	2.5%	HGGVRLY	1.3%	YVTRTPY	1.5%
HTNSAYI	1.7%	HYIDFRW	1.2%	VSYGVPM	0.8%
GMMSSPP	1.6%	ATKHYTT	1.0%	HYIDFRW	0.7%
DAIPTSV	1.5%	AVDPQYG	1.0%	KGIYHQV	0.3%
HAGFVPS	1.4%	DAIPTSV	0.8%	AVDPQYG	0.3%

CONTROL: C1					
EFARNSI	21.6%	EFARNSI	78.7%	EFARNSI	75.7%
LDIVDAP	3.6%	QHDTVPP	6.7%	HYIDFRW	18.7%
ALPLLDA	2.6%	HSTQLPY	3.5%	QHDTVPP	1.6%
FSTNFGN	2.5%	HYIDFRW	2.2%	FVPHKWY	1.1%
QHDTVPP	2.1%	FVPHKWY	1.9%	YGNSGIV	1.0%
HISGRPL	2.0%	AMSHSKQ	1.2%	AMSHSKQ	0.8%
VNGHYTI	2.0%	FCPDCKP	0.8%	FCPDCKP	0.4%
QIGHDGN	1.8%	TGKMLAD	0.6%	HSTQLPY	0.1%
GQAMNHT	1.8%	GAAYIRA	0.5%	NVAHKMF	0.1%
DFHPDVL	1.7%	NVAHKMF	0.5%	TGKMLAD	0.0%

CONTROL: C2					
VSRDTPQ	28.4%	VSRDTPQ	79.0%	VSRDTPQ	80.3%
SYTPGGH	16.7%	SAEKLLR	6.9%	SAEKLLR	13.2%
LIQPITT	7.4%	YNISVNK	3.0%	GQSEKHL	3.5%
QLYREFN	4.2%	QLYREFN	2.4%	YNISVNK	1.1%
MDTPDRI	3.5%	WVTDSSW	1.7%	WVTDSSW	0.8%
WVTDSSW	3.2%	HSPNSIK	1.4%	QLYREFN	0.4%
HSPNSIK	2.9%	GQSEKHL	1.3%	LPGNRLL	0.3%
RFDWSYP	2.9%	RFDWSYP	1.2%	RFDWSYP	0.3%
AYASDSY	2.7%	LPGNRLL	0.9%	HSPNSIK	0.0%
YNISVNK	2.6%	RTWEPYT	0.5%	RTWEPYT	0.0%

CONTROL: C3					
SNYHPSI	51.2%	SNYHPSI	61.0%	AERYPDS	56.8%
TTQVLEA	3.1%	KMISATE	6.2%	SNYHPSI	27.6%
KMISATE	2.9%	AERYPDS	5.2%	KMISATE	8.0%
LPQSWAM	1.8%	KMESGTA	3.7%	ELGTTQT	2.3%
ATAEWHP	1.7%	AKYEAGS	3.2%	TTQVLEA	1.0%
APANTFT	1.7%	TTQVLEA	1.9%	AKYEAGS	0.7%
VPPTEHP	1.3%	TEQKIEA	1.9%	GWETRME	0.5%
LGQLFPQ	1.3%	TITAMQG	1.4%	NDRPHMP	0.3%
MIHPRDQ	1.1%	AERILSV	1.3%	ADLMHFT	0.3%
FTQPLYK	1.1%	GWETRME	1.2%	VPPTEHP	0.3%

CONTROL: C4					
KSLNSTI	17.2%	VPILNSL	19.8%	WSLSELH	20.6%
VPILNSL	7.1%	WSLSELH	17.0%	HAAWHFI	19.3%
SMNETFA	6.6%	ENHVHVR	13.2%	ENHVHVR	16.7%
WSLSELH	3.7%	QETRGTY	4.8%	VPILNSL	7.6%
NTDTTRE	3.3%	IDNSHTH	4.0%	VTLPPSS	3.8%
VNQSWDL	2.0%	TTANVRI	3.9%	TTANVRI	3.4%
RDEVYFH	1.9%	VNQSWDL	3.1%	ESKWMPL	3.2%
NEAPRHA	1.7%	HAAWHFI	2.9%	IDNSHTH	3.2%
IDRTQFM	1.5%	EPAIATP	2.3%	QETRGTY	3.0%
NTVGTVQ	1.3%	NEAPRHA	2.3%	GTMHSMK	2.6%

CONTROL: C2p					
MGQPTVK	58.0%	QQLAVDT	39.8%	QQLAVDT	64.5%
GPLKTWK	18.6%	MGQPTVK	16.2%	ESRVMSR	14.4%
GTLASLN	5.4%	TTQVLEA	8.8%	WEGPQPK	7.9%
KVKTYDM	3.4%	WEGPQPK	6.9%	ALGTRMI	6.5%
QQLAVDT	2.7%	GPLKTWK	6.8%	STLNWWN	2.7%
STLNWWN	1.4%	ESRVMSR	4.1%	KDNTYVM	1.0%
QTNMRAP	0.4%	GTSDKLP	2.3%	TTQVLEA	0.8%
ESRVMSR	0.3%	KDNTYVM	2.3%	GTSDKLP	0.5%
EARMHRA	0.3%	STLNWWN	2.1%	HSHTLTW	0.4%
TYPSLWK	0.3%	GTLASLN	1.3%	YPWFIRA	0.3%

CONTROL: C3p					
DVHKLGN	23.8%	DVHKLGN	37.4%	AQSLETS	40.5%
ISHTKAE	10.0%	AQSLETS	12.9%	ETALIAA	16.6%
NDIPHLM	9.1%	NDIPHLM	12.3%	QQLAVDT	11.0%
RIHVHNS	4.4%	ETALIAA	7.5%	DVHKLGN	10.8%
AQSLETS	3.5%	QQLAVDT	3.8%	LYADSVL	7.7%
SLTMKAN	2.1%	RIHVHNS	3.5%	RIHVHNS	3.1%
DLNMPIS	2.0%	LYADSVL	3.2%	NDIPHLM	2.8%
LYADSVL	1.9%	SVASTTV	3.2%	NPMYHSS	1.9%
ITPAYDN	1.8%	DLNMPIS	2.1%	KQEAMTL	1.1%
SVASTTV	1.7%	IAGPFKL	1.6%	QNSPFSE	1.0%

Supplementary Table S5. The ten most frequent peptides and their frequencies for each sample. The peptides are separated according selection round (Round 1-3). The peptides with a grey overlay were also observed in a control sample with a prevalence > 1.

Peptide	# of samples		Peptide	# of samples
QLYREFN	11		AERYPDS	3
SLLGQTP	8		DSSLFAL	3
TTQVLEA	7		DSSSFAL	3
VSRDTPQ	7		DTALHSL	3
IDRTQFM	7		EFARNSI	3
WSLSELH	7		GGPSGKL	3
YPWFIRA	7		GLAPFNA	3
DAIPTSV	6		GQSEKHL	3
KMISATE	6		GWETRME	3
NEAPRHA	6		IRVPPLV	3
AQYVAVG	5		KFAASSY	3
DGRYYIN	5		LSKINSP	3
DVHKLGN	5		LVIHSRT	3
ENHVHVR	5		LYADSVL	3
SNYHPSI	5		NEHTGNL	3
AQSLETS	5		NPPWFHT	3
DALAATL	5		NVRYNMI	3
DNYDSTA	5		QQLAVDT	3
GLRNPPS	5		QQTNWSL	3
IDNSHTH	5		SAAWNKS	3
TTANVRI	5		STPATLI	3
TNESYRH	4		TNLSHVP	3
TVISQNM	4		VPILNSL	3
EMIYEAN	4		VVTPKTA	3
QLSLIST	4		YYNTTPN	3
STIVAEM	4		ALGTRMI	3
DLNMPIS	4		EVMGRLA	3
GTGSQAS	4		GASESYL	3
GTSIYLH	4		GGSTDCI	3
HGGVRLY	4		GHDPTPL	3
HLNQQNH	4		LAKPLLV	3
HYIDFRW	4		LSANHWV	3
IHMPASP	4		MHTVAVQ	3
SIMNDRY	4		MPTWLHH	3
AYVARQN	4		SQPTWMF	3
KHLTAMA	4		SYTDLLR	3
SHVNVPS	4		THNLPVV	3
SSNQFHQ	4		TLSERNI	3
DSMSLLQ	3		TVTFIDS	3
HWSDDPH	3		VSGLLVE	3
SDRVLDN	3		YSLKQYQ	3

Supplementary Table S6. Dominating TUP candidates. Peptides that have been identified with a peptide prevalence >1 in a minimum of 3 different samples, including at least 1 control sample, are listed. The number of samples were a given peptide was observed is listed in the “# of samples” column.

Round 2	Round 3
IWRLPTH	IWRLPTH
WRLPYHN	HWRLPLH
WRHPSHF	WRLPYHN
WRLPYHN	WRHPSHF
TWRHPHH	WRHPSHF
HWRLPLH	TWRMPYH
TWRMPYH	WRLPYHN
QWQRPMH	TWRHPHH
WRLPYHP	VWRLPTH
WRLPEHR	QWQRPMH
IWRLPLH	WRLPYHN
LRLGPTH	TWKHPHH
WSLPELH	WWHPSHF
WRHPTHD	WRLPEHR
QWQRPMH	WRHPTHD
HWRLPTH	TWRMPYH
WLRLSTH	IWRMPTH
HWRLPLH	
HWQLPLH	
IWRPAAV	
AWRLPLH	
WRYPSHF	
WRMPLHR	
WRDPSHF	
IWRLPPQ	
WRHPSHF	
TWRMPYH	
IWRLQTH	

Supplementary Table S7. List of peptides in the significant peptide clusters separated according to selection round.

	Round 2	Round 3
**P1**	13	8
**P1_12**	4	3
**P2**	0	0
**P3**	4	4
**P4**	2	0
**P4_12**	2	1
**Controls**	3	1
**Combined**	28	17

Supplementary Table S8. The number of peptides in the significant clusters in selection round 2 and 3 separated according to the patient sample they were derived from.

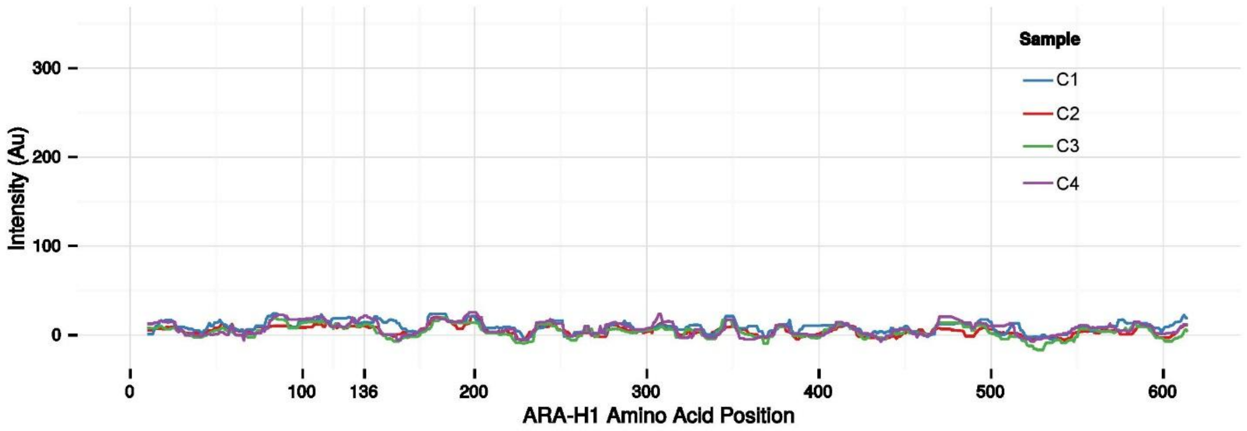

Supplementary Figure S4. Intensity based on control IgE reactivity to Ara h 1 as measured by peptide micro-arrays. The right-bound rolling median (window size 12) of the mean intensity of the triplicate 12-mer peptides overlapping each residue is shown for every control sample. The start position of the epitope identified in the patients, at position 136, has been specifically marked.

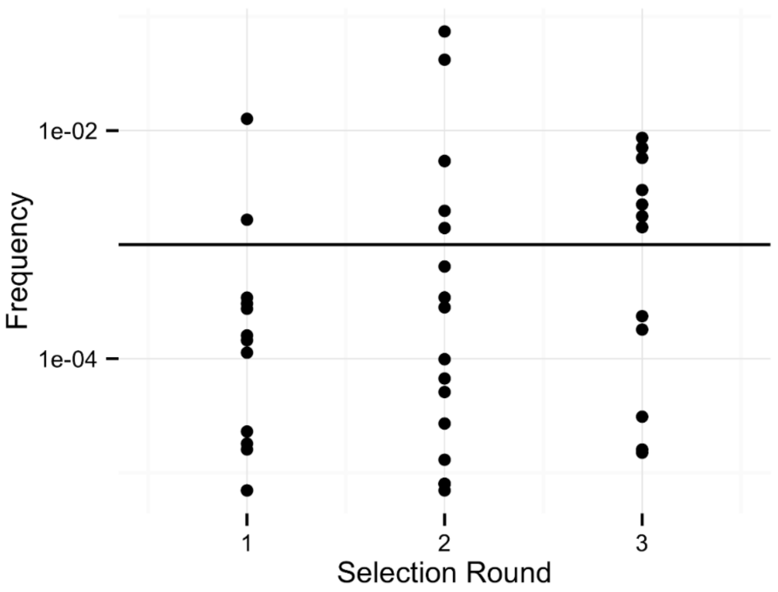

Supplementary Figure S5. Frequency of peptides that were included in the significant cluster. The logarithmic peptide frequencies are separated according to selection round (1-3). The horizontal line represents the suggested detection limit of traditional phage display approaches, assuming that 1000 phage colonies are assayed thereby detecting peptides with a frequency above 0.001. Peptides with a rank score of 0 have not been plotted.
